# Development and Validation of a Core Literacy Scale for Physical Education Among Chinese University Students

**DOI:** 10.3390/bs15121655

**Published:** 2025-12-02

**Authors:** Yimai Wu, Chaojie Wang, Hong Liu, Chunyan Zhang, Yasi Jin, Ti Hu, Hang Min

**Affiliations:** College of P.E. and Sports, Beijing Normal University, Beijing 100875, China; 202422070038@mail.bnu.edu.cn (Y.W.); 202521070006@mail.bnu.edu.cn (C.W.); 202431070009@mail.bnu.edu.cn (H.L.); 202321070017@mail.bnu.edu.cn (C.Z.); 202321070002@mail.bnu.edu.cn (Y.J.)

**Keywords:** core literacy, physical education, scale development, validation, reliability, university students

## Abstract

Background: Cultivating core literacy in physical education (PE) is increasingly recognized as crucial for talent development in higher education. Although diverse assessment tools exist for various literacies, none specifically evaluate the core literacy of the PE discipline among Chinese university students. This study aimed to develop and validate a scale for assessing this core literacy. Methods: An initial item pool was developed through a comprehensive literature review and in-depth expert interviews. Following three rounds of Delphi expert consultation (*n* = 15) and a pilot test, five items exhibiting insufficient discriminative power or conceptual redundancy were removed, yielding the final Chinese University Students’ Physical Education Core Literacy Assessment Scale. The scale was administered to 1008 university students. Its reliability and validity were examined through exploratory factor analysis (EFA) and confirmatory factor analysis (CFA). Results: The finalized scale contains 43 items across three dimensions. Model fit indices were excellent: χ^2^/df of 1.252, RMSEA of 0.016, CFI of 0.934, TLI of 0.992. Conclusion: The newly developed scale proves to be a valid and reliable instrument for measuring core PE literacy among Chinese university students.

## 1. Introduction

In recent years, the concept of Core Literacy has emerged as a key trend in global education reform (Reimers, 2021). It constitutes a coherent framework spanning multiple domains, comprehensively addressing diverse aspects of college students’ lives. Within this framework, competencies related to tool usage for both individual and social development are accorded equal importance (Kowalewska, 2016), representing a central focus in contemporary educational research and practice. Current scholarship on Core Literacy is largely shaped by several influential frameworks: first is the “Framework for Core Literacy” proposed by the Organization for Economic Cooperation and Development (OECD) aiming at achieving the goals of successful lives and the development of a sound society (Y. C. Chen et al., 2023); second is the “key competences indicators” constructed by the EU, which are designed to promote lifelong learning (Castillo et al., 2011); thirdly, there is the “21st Century Skills Framework” represented by countries like the United States, Japan, and Singapore, which centers on workplace requirements (Faraniza, 2021); fourthly, taking the literacy framework in the Asian region as an example, emphasizes the importance of core values (Tabrani et al., 2024); fifth is the framework system represented by Russia, which focuses on the quality of citizens’ daily life and cultural leisure (Romashko, 2020). The OECD proposed three essential criteria for defining key competencies in 2005 (Paakkari & Okan, 2019): ① they must benefit positive outcomes for both society and individuals; ② they must help individuals to meet key needs across diverse contexts; ③ they must extend significantly beyond specialists in specific domains to be crucial for all people. This global consensus on the importance of key competencies underscores their role as a pivotal measure of individual quality of life and societal advancement.

Although the specific term “Core Literacy” is absent from ancient Chinese classics, its underlying principles are deeply embedded in traditional educational philosophy and culture. Confucianism, which has long dominated Chinese education, emphasizes virtue cultivation (Gu, 2006). Ancient sages were dedicated to determining “what kind of talents to cultivate.” Informed by this heritage and China’s social realities, the “Core Literacy for the Development of Chinese Students” framework systematically constructs a quality requirement system for talent development. It explicitly defines Core Literacy as the essential qualities and key abilities students need to adapt to lifelong development and societal progress.

The Core Literacy framework in Chinese Physical Education and health is inherently localized. It is theoretically derived from the national “System of Core Literacy for Chinese Students’ Development,” constituting a disciplinary application and realization of this broader mandate. As a foundational pillar for talent cultivation (Zhao & Zhang, 2020), the assessment of college students’ Core Literacy is of paramount importance for driving the high-quality development of higher education. University physical education plays a critical role in promoting well-rounded development, which integrates moral, intellectual, physical, esthetic, and labor education. Therefore, the cultivation of Core Literacy within physical education is fundamental to the holistic development of college students (Zhang et al., 2022).

Internationally, particularly in regions such as the United States and Europe, research often adopts “sports literacy” (Fang & Zhang, 2024) or “physical literacy” (She et al., 2023) as its conceptual foundation. A widely used instrument in this context is the “Perceived Sports Literacy Scale,” designed to evaluate the sports literacy of physical education teachers (Sum et al., 2016). However, these concepts are distinct from China’s Core Literacy in PE. Physical literacy, for instance, is defined as the combination of motivation, confidence, physical skills, knowledge, and understanding required to maintain and enhance physical activity throughout life (Whitehead, 2001). In contrast, Core Literacy in PE is cultivated through deliberate scientific instruction and effective pedagogical practices. Its development is recognized as vital for fostering students’ all-around development and equipping them to meet the demands of modern society (Edwards et al., 2017). Within the Chinese context, the teaching of PE Core Literacy at the university level is often not explicitly evaluated, creating a gap between ambitious curricular goals and actual assessment practices. While national curricula emphasize movement competence, health behavior, and sports morality, teaching and assessment in practice frequently prioritize discrete sports skills and physical performance metrics (Shao et al., 2020). This discrepancy between the holistic educational objectives and the narrow focus of prevailing assessment methods highlights the critical need for a tailored instrument. Consequently, directly applying international physical literacy scales is inadequate for measuring the multifaceted construct of PE Core Literacy among Chinese university students.

Current evaluation practices in university physical education remain limited in their capacity to assess the comprehensive development of students. These limitations include an overemphasis on discrete skill acquisition rather than holistic development (X. Li & Zhang, 2022), a narrow focus on motor performance instead of broader literacy cultivation (Wang & Liu, 2022), and a reliance on teacher-centered assessment approaches that neglect student self-evaluation and peer feedback (L. Li & Dapat, 2023). Underlying these practical shortcomings is a fundamental conceptual ambiguity regarding what constitutes “core literacy” in physical education and how it differs from related international concepts.

As students mature cognitively and socially, their involvement in physical education must evolve from basic skills to higher-order capacities like sports culture inheritance, scientific inquiry, and social participation (Brown et al., 2017). The Chinese framework is uniquely positioned to foster these advanced competencies through its comprehensive structure. This fundamental conceptual divergence clarifies why assessments designed for “physical literacy” are inherently limited in evaluating the multifaceted outcomes of the Chinese core literacy framework, thereby necessitating the development of a contextually appropriate instrument.

## 2. Materials and Methods

### 2.1. Research Framework and Conceptual Definitions

This study is grounded in the conceptual framework of The Physical Education and Health Curriculum Standards for Compulsory Education (2022 Edition) (Ji, 2022), which defines the three core constructs as follows: Movement competence is defined as the integrated ability students demonstrate during sports participation. This construct comprises three dimensions: physical fitness, cognitive understanding and application of techniques and tactics, and competitive performance. Health behavior refers to students’ holistic conduct in enhancing physical and mental well-being while actively adapting to their environment. It is characterized by four dimensions: awareness and habits of physical exercise, mastery and application of health knowledge, emotional regulation, and environmental adaptation. Sports morality encompasses the behavioral norms, ethical principles, and valued spiritual qualities students develop in sports. This concept is manifested through three dimensions: sportsmanship, sports ethics, and sports character. This study aimed to precisely define the structural dimensions of university students’ PE Core Literacy, building upon a theoretical foundation that incorporates prior work on concepts like “physical literacy.” To this end, a rigorous scale development process was undertaken to create and validate a tailored instrument for the Chinese educational and cultural context.

### 2.2. Scale Development: Item Generation and Expert Review

Building upon the theoretical framework and prior research discussed in the introduction, and supplemented by semi-structured interviews, this study sought to identify the structural dimensions of university students’ Physical Education Core Literacy. The development of the initial item pool was guided by an existing research framework and involved the systematic adaptation and integration of items from well-established scales. To ensure the scale’s content validity and scientific rigor, a panel of 15 experts was invited to evaluate the items in terms of semantic clarity, appropriateness, and dimensional relevance. Experts were selected through purposive sampling based on the following criteria: ① possession of a doctoral degree and the academic rank of associate or full professor; ② at least five years of research experience in core literacy, physical education, or related disciplines; ③ representation across key fields, including education, psychology, physical education, and physiology. Over three rounds of Delphi consultation, the item wording, structure, and formatting were systematically refined. The expert panel also formally assessed the content validity of the preliminary scale.

Based on the synthesis of qualitative expert feedback and quantitative pilot test data, the scale refinement followed established psychometric procedures (Colbert et al., 2019). The expert questionnaire was specifically designed to assess content validity, comprising two main parts: experts’ basic information and a five-point Likert scale evaluating each item in terms of semantic appropriateness, dimensional fit, and overall relevance. The revision process systematically integrated multiple sources of evidence:

(1) For item refinement, expert evaluations of semantic clarity and dimensional alignment were cross-validated with statistical results from item analysis. For instance, the Movement Competence item was rephrased from “I possess the competitive physical fitness…” to “I have the ability to engage in physical fitness training…” to better reflect trainable capabilities, following expert recommendations supported by pilot data.
(2) Dimensional reorganization was guided by both conceptual coherence and statistical patterns. Three Health Behavior items addressing sports injury management, venue hygiene practices, and infectious disease prevention were relocated to Movement Competence based on expert assessment of their better alignment with practical activity implementation, which was further confirmed through factor loading patterns in pilot analysis.
(3) Item consolidation was conducted through content analysis and statistical examination. Health Behavior items 11 and 12 were merged into a single comprehensive statement—“I will choose appropriate physical activities to alleviate negative emotions and stress…”—after both expert evaluations identified semantic redundancy and statistical analysis revealed high inter-item correlation.

These systematic revisions, integrating Delphi method outcomes with psychometric analysis, resulted in the Preliminary Scale of Core Literacy in Physical Education for Chinese University Students, comprising 3 dimensions and 48 items measured on a 5-point Likert scale from “Very Inconsistent” to “Very Consistent”.

### 2.3. Pilot Testing and Item Refinement

A systematic scale development procedure was followed, beginning with a 48-item preliminary questionnaire. In accordance with psychometric sampling recommendations (Colbert et al., 2019), the pre-test was administered via Wenjuanxing—a professional online survey platform prevalent in Chinese academic research—to 520 undergraduate students at a university branch campus in Beijing. This yielded 506 valid responses, corresponding to a high effective response rate of 97.3%.

The pilot data underwent a comprehensive validation protocol, including assessments of content validity, item analysis, and preliminary checks of reliability and validity. These quantitative results, complemented by qualitative feedback from field research, informed the refinement of the scale through item revision and deletion.

To enhance the sample’s representativeness and demographic balance for the main study, a stratified random sampling strategy was employed across diverse tiers of Beijing universities, including 985 Project, 211 Project, and Double First-Class institutions. Methodological refinements based on the pilot study involved clarifying specialized terms, optimizing Likert scale anchors, and improving the informed consent process. The subsequent validation of the finalized scale using advanced statistical techniques, namely Exploratory Factor Analysis (EFA) and Confirmatory Factor Analysis (CFA), is presented in Section 3.

### 2.4. Item Analysis and Preliminary Reliability

Item analysis refers to the process of systematically analyzing data collected through questionnaires, aiming to extract information from the data regarding whether the quality of the questionnaire is acceptable and whether any items should be deleted (X. J. Chen et al., 2024). The procedure involved: ① calculating the total scale score for each participant; ② dividing participants into high-score (top 27%) and low-score (bottom 27%) groups based on total scores; ③ performing independent samples *t*-tests for each item between the high and low groups. Items demonstrating a statistically significant difference (*p* < 0.05) were retained. In this experiment, 506 valid sample data were first collected, and item analysis was conducted on these data to test the quality of the questionnaire. Additionally, Cronbach’s alpha coefficient was calculated to assess the internal consistency reliability of the scale dimensions using the pilot data. The alpha coefficients were 0.932, 0.915, and 0.929, all exceeding the recommended threshold of 0.7, indicating good preliminary reliability for the pilot scale dimensions.

## 3. Results

### 3.1. Participants and Final Scale Administration

Following the promising reliability of the pre-test, the main validation was undertaken via a cross-sectional design. The finalized 43-item PE Core Literacy scale was administered, yielding a final sample of 1008 valid questionnaires. The participant demographic profile is presented in Table 1. Briefly, the sample comprised 496 males (49.2%) and 512 females (50.8%). Academic discipline representation spanned Science (15.18%), Literature (18.75%), Law (12.80%), Engineering (15.28%), Management (10.62%), Art Studies (13.69%), Philosophy (11.81%), and other fields (1.88%). Students were distributed across Freshman (23.81%), Sophomore (27.38%), Junior (23.31%), and Senior (25.50%) years. All participants met the following inclusion criteria: age between 18 and 24, full-time undergraduate enrollment, voluntary informed consent, absence of physical or psychological impairments, and the capability to complete the survey independently.

### 3.2. Reliability Analysis

The internal consistency reliability of the final scale dimensions was assessed using Cronbach’s alpha (Kline, 2013). As presented in Table 2, all dimensions demonstrated excellent reliability, with coefficients significantly exceeding the established threshold (>0.7).

### 3.3. Exploratory Factor Analysis

To validate the factor structure of the PE Core Literacy scale, a two-step factor analysis approach was implemented. The following sections present the empirical results of these analyses, which support the hypothesized three-dimensional model.

#### 3.3.1. Verification of Data Suitability for Factor Analysis

Prior to conducting exploratory factor analysis, the suitability of the data was assessed. This study employed the Kaiser-Meyer-Olkin (KMO) sampling adequacy test and Bartlett’s test of sphericity for evaluation. As shown in Table 3, the KMO value was 0.982 (excellent, >0.6), and Bartlett’s test was highly significant (χ^2^ of 31,814.127, df of 903, *p* < 0.001). These results confirm the data’s suitability for factor analysis.

#### 3.3.2. Factor Extraction (Initial Eigenvalues and Variance Explained)

To examine the structural validity of the scale, Exploratory Factor Analysis (EFA) was performed based on the correlation matrix. Factors were extracted using Principal Component Analysis and rotated via the Varimax orthogonal rotation method to achieve a clearer and more interpretable factor structure. Factor extraction adhered to the Kaiser criterion, retaining three factors with eigenvalues greater than 1, which was consistent with the hypothesized three-dimensional model. These three factors collectively accounted for 61.88% of the total variance, exceeding the conventional threshold of 60%. This indicates that the extracted factors sufficiently capture the majority of the information contained within the original 43 items. Specifically, prior to rotation, the first factor explained 38.74% of the variance. Following rotation, the variance contribution was redistributed, resulting in a more balanced explanatory power across the three factors (24.86%, 19.98%, and 17.05%, respectively), which further refines the interpretability of the factor solution. In summary, the results of the factor analysis support the robust structural validity of the scale (Table 4).

#### 3.3.3. Rotated Factor Loadings

An exploratory factor analysis using principal component analysis with varimax rotation yielded a clear three-factor solution (converged in 6 iterations). As presented in Table 5, all items loaded significantly (loadings > 0.60; range: 0.669–0.908) on their intended factors, with no notable cross-loadings. The structure precisely matched the theoretical dimensions: Factor 1 (Health Behavior, items B1–B17), Factor 2 (Sports morality, items C1–C14), and Factor 3 (Athletic Competence, items A1–A13). These results robustly support the scale’s construct validity and the stability of the three-factor model.

### 3.4. Confirmatory Factor Analysis (CFA)

Confirmatory Factor Analysis (CFA) was conducted using AMOS to examine the scale’s convergent and discriminant validity. Average Variance Extracted (AVE) and Composite Reliability (CR) were calculated for each dimension based on standardized factor loadings. Convergent validity was considered adequate if AVE ≥ 0.5 and CR ≥ 0.7 (Fornell & Larcker, 1981).

#### 3.4.1. Convergent Validity

Composite Reliability (CR), a key indicator of latent variable reliability, is primarily used to assess the consistency between latent variables and their observed indicators within the measurement model (Rahman & Rahman, 2023). When the standardized factor loadings of observed variables exceed 0.5 and CR values are higher than 0.6, the measurement model can be determined to have good internal consistency (Kim, 2023). The magnitude of CR values directly reflects the strength of the inherent association between latent variables and their measurement indicators. Data analysis indicates that the psychometric indicators for all three dimensions meet the criteria: (1) the average variance extracted (AVE) for each dimension is >0.5, with CR values all >0.7; (2) the standardized factor loadings for all items are >0.6. These results collectively indicate that the measurement model exhibits good convergent validity, with each observed variable effectively reflecting the characteristics of its corresponding latent variable.

#### 3.4.2. Discriminant Validity

After validating the convergent validity, the AVE-correlation coefficient comparison method was employed to test the discriminant validity (Zulfiqar et al., 2018). As presented in Table 6, the square root of the AVE (diagonal, bolded) for each dimension was greater than its correlations with all other dimensions (off-diagonal values), satisfying the Fornell–Larcker criterion and confirming good discriminant validity.

#### 3.4.3. Model Fit Indices

The overall model fit was evaluated using multiple indices (Browne & Cudeck, 1992; Hu & Bentler, 1999). As shown in Table 7, the model demonstrated excellent fit to the data: χ^2^/df of 1.292 (<3), RMSEA of 0.017 (<0.08), CFI of 0.952 (>0.90), TLI of 0.992 (>0.90), IFI of 0.992 (>0.90). All indices met or exceeded recommended thresholds, indicating very good model fit and supporting the scale’s structural validity.

## 4. Discussion and Conclusions

The development and validation of the Core Literacy Scale for Physical Education for Chinese University Students represents a critical step toward bridging the gap between the theoretical emphasis on holistic development and the practical need for its assessment in higher education. Our findings, derived from a rigorous scale development process, confirm the multidimensional nature of this construct, which comprises movement competence, healthy behavior, and sports morality. This three-factor structure not only aligns with the national “Core Literacy for Chinese Students’ Development” framework but also establishes a clear conceptual and operational distinction from internationally prevalent constructs like “physical literacy.”

While international research has significantly advanced our understanding of physical competence and motivation, the salient emergence of sports morality as a core dimension underscores the profound influence of the Confucian educational tradition. This finding validates the need for a culturally contextualized instrument, as directly applying international physical literacy scales may overlook this fundamental aspect of development in China. The scale’s excellent psychometric properties provide strong evidence that this construct can be measured with precision and consistency.

This study addresses a key limitation in current university physical education evaluation, which has often been criticized for its narrow focus on discrete motor skills and physical performance metrics. Furthermore, the integration of healthy behavior moves the assessment beyond physical prowess towards a more holistic view of student well-being, resonating with global perspectives on comprehensive health.

The implications of this work are threefold: Theoretically, it operationalizes a key national educational framework within the physical education domain, providing a measurable model for future research on holistic education. Practically, the scale offers educators and policymakers a reliable tool to move beyond skill-based assessment, enabling the evaluation of broader educational goals and serving as a diagnostic tool to refine curricula and teaching strategies toward literacy-centered education. Regarding wider applicability, the core tripartite structure of our scale—encompassing competence, behavior, and morality—may provide a valuable template for researchers in other cultural contexts, particularly those that also emphasize moral and social development. Future studies could adapt this model to explore its cross-cultural validity, investigating how the manifestation of each dimension varies across different national settings.

In conclusion, this study develops and validates the first instrument designed to assess physical education core literacy in Chinese universities. By offering a reliable and valid tool, it bridges the gap between national educational theory and practice. Consequently, this work contributes to international efforts to redefine educational success, facilitating a meaningful transition from skill-based instruction to an education centered on fostering well-rounded, resilient, and ethically grounded individuals.

## 5. Limitations and Future Directions

Although the scale developed in this study demonstrates good reliability and validity, several limitations warrant in-depth exploration in future research:

First, geographical coverage was limited. Although the sample size was adequate, participants were recruited solely from universities in Beijing. Future studies should include samples from broader geographical regions and diverse types of institutions to further enhance the scale’s universality and representativeness.

Second, validity evidence could be expanded. While internal consistency and construct validity were established, evidence for test–retest reliability (stability) and criterion validity is lacking. Future research could examine the scale’s stability through longitudinal designs and strengthen validity support by correlating results with actual behavioral indicators.

Third, demographic variables warrant further refinement. While differences by academic background were noted, other potential factors (e.g., socioeconomic status, urban/rural origin, sports resource access) were not sufficiently explored. Future research should incorporate these variables to provide evidence for educational equity policies.

## Figures and Tables

**Table 1:** Participant Demographic Characteristics (*n* = 1008).

Variables	Category	*n*	%
Gender			
	Male	496	49.3%
	Female	512	50.7%
Student degree major			
	Science	153	15.18%
	Literature	189	18.75%
	Law	129	12.80%
	Engineering	154	15.28%
	Management	107	10.62%
	Art Studies	138	13.69%
	Philosophy	119	11.81%
	Others	19	1.88%
Grade			
	Freshman	240	23.81%
	Sophomore year	276	27.38%
	Junior year	235	23.31%
	Senior year	257	25.50%

**Table 2:** Internal Consistency Reliability (Cronbach’s Alpha) by Dimension.

Dimension	Number of Items	Cronbach’s Alpha
Movement competence	12	0.939
Healthy behavior	18	0.960
Sports morality	13	0.959

**Table 3:** KMO and Bartlett’s Test of Sphericity.

Category	Item	Value
Sampling Adequacy	KMO	0.982
Bartlett’s Spherical test	Chi-square approximation	31,814.127
	DF	903
	*p*-value	0.000

**Table 4:** Total Variance Explained by Extracted Factors.

Factor	Initial Eigenvalues		Rotation Sums of Squared Loadings	
	Eigenvalue	Variance %	Variance %	Cumulative %
1	16.66	38.74	24.86	24.86
2	6.43	14.96	19.98	44.83
3	3.52	8.18	17.05	61.88

**Table 5:** Rotated Factor Loadings (Pattern Matrix).

Item	Factor 1 (Healthy Behavior)	Factor 2 (Sports Morality)	Factor 3 (Movement Competence)
A1 ^1^			0.876
A2			0.713
A3			0.727
A4			0.714
A6			0.714
A7			0.722
A8			0.726
A9			0.748
A10			0.669
A11			0.712
A12			0.72
A13			0.723
B1 ^1^	0.908		
B2	0.747		
B3	0.727		
B5	0.742		
B6	0.743		
B7	0.746		
B8	0.724		
B9	0.737		
B10	0.75		
B11	0.739		
B12	0.734		
B13	0.715		
B14	0.743		
B15	0.757		
B16	0.733		
B17	0.743		
C1 ^1^		0.915	
C2		0.743	
C4		0.762	
C5		0.759	
C6		0.731	
C7		0.758	
C8		0.775	
C9		0.757	
C10		0.764	
C11		0.772	
C12		0.749	
C13		0.75	
C14		0.736	

^1^ Factor loadings below 0.40 are suppressed for clarity. Item codes (A-series: Movement Competence; B-series: Healthy Behavior; C-series: Sports Morality) reflect the final scale structure, and omitted indices (e.g., A5, B4, C3) correspond to items deleted during scale refinement.

**Table 6:** Discriminant Validity Assessment: Fornell–Larcker Criterion (Square Root of AVE on Diagonal).

	Sports Morality	Healthy Behavior	Movement Competence
Sports morality	0.802		
Healthy behavior	0.401	0.755	
Movement competence	0.560	0.365	0.752

**Table 7:** Confirmatory Factor Analysis Model Fit Indices.

Indicator	χ^2^	df	χ^2^/df	RMSEA	TLI	CFI	IFI
Standard	-	-	<3	<0.08	>0.9	>0.9	>0.9
value	1107.306	857	1.292	0.017	0.992	0.952	0.992

## Data Availability

The data that support the findings of this study are available from the corresponding author upon reasonable request.
